# Books and Magazines

**Published:** 1914-02

**Authors:** 


### Books and Magazines.

Disease and Its Causes. W. T. Councilman, M. D., LL. D., Professor of Pathology, Harvard University. New York. Henry & Co. Price 50 cents.

This is No. 68 of the Home University Library of Modern Knowledge.

The author says in his preface that the book is not written for physicians, and immediately adds: "It has been assumed that the reader has some familiarity with elementary anatomy and physiology." [Few lay people have.] He has "endeavored to portray disease as life under conditions which differ from the usual," and proceeds to discuss histology, cells as living units, amoeba as type of a unicellular animal; in fact, goes into the subject very deeply—over the head of the average non-professional reader. There are a number of illustrations; one a longitudinal section through the middle of the body, showing the external and internal surfaces and the organs. It is a much more pretentious book than it appears to be, and is worthy of careful study by anyone, lay or professional.

---

A Manual of X-Ray Technic. By Arthur C. Christie, Captain Medical Corps U. S. Army; Instructor in Radiology and Operative Surgery, Army Medical School, Washington, D. C. With 42 illustrations. J. B. Lippincott Company, Philadelphia. Price $2.00.

This manual has been prepared to meet the needs of the medical service of the United States army, and the author says it has been his aim to limit it to the absolute essentials, a knowledge of which will enable the operator to do satisfactory work in X-ray diagnosis.

It will be found useful for these purposes by many physicians in private practice who prefer to do their own X-ray work, and especially by young doctors. We recommend our readers to buy it.

---

Stammering and Cognate Defects of Speech. By C. S. Bluemel. 2 vols. Price $5.00 net. Published by G. E. Stechert & Co., New York, London, Leipzig and Paris, 1913. Vol. 1, The Physchology of Stammering, 365 pages. Vol. 2, Contemporaneous Systems of Treating Stammering: Their Possibilities and Limitations. 390 pages including glossary and copious index.

This is quite a pretentious work, and goes deeply into the subject. It should have a wide sale, but the unfortunates have been so often imposed upon by pretenders that they are a little shy. I do not know who the author is; it seems he is a layman, and lives at Boulder, Colorado. It is a very learned treatise, and I suppose there is no doubt that it is authoritative. It makes excellent smooth reading.

